# The correlation between the serum LDL-C/Apo B ratio and lumbar bone mineral density in young adults

**DOI:** 10.1186/s12891-023-06325-w

**Published:** 2023-03-22

**Authors:** Anjun Tan, Juntao Shu, Hong Huang, Heng Shao, Jingjing Yang

**Affiliations:** 1 Department of Geriatrics, The First People’s Hospital of Yunnan Province, The Affiliated Hospital of Kunming University of Science and Technology, Kunming, Yunnan 650034 People’s Republic of China; 2 Department of Neonatology, The Children’s Hospital of Kunming City, The Affiliated Children’s Hospital of Kunming Medical University, Kunming, Yunnan 650103 People’s Republic of China

**Keywords:** Serum low-density lipoprotein cholesterol/apolipoprotein B ratio, Bone mineral density, NHANES, Cross-sectional study

## Abstract

**Background:**

Numerous studies have confirmed that atherosclerosis is related to osteoporosis (OP), and patients with atherosclerosis are more prone to OP. The ratio of low-density lipoprotein cholesterol (LDL-C) to apolipoprotein B (Apo B) is the valid indicator of atherosclerosis. Nevertheless, conclusions regarding relation between LDL-C/Apo B ratio and bone mineral density (BMD) are still lacking. As a result, this study concentrated on investigating the relationship between LDL-C/Apo B ratio and lumbar BMD in the young adult population according to the National Health and Nutrition Examination Survey (NHANES).

**Methods:**

Information of 2027 young adults (age 20–40 years) from NHANES database was obtained for this cross-sectional study. The correlation between serum LDL-C/Apo B ratio and lumbar BMD was explored through weighted multiple stratified linear regression, while the smooth curve fitting model was utilized for analyzing nonlinear relation. In the nonlinear relation, the inflection point was calculated by saturation threshold analysis. The weighted two-piecewise linear regression model was constructed.

**Results:**

After covariates were adjusted, the relation between serum LDL-C/Apo B ratio and lumbar BMD varied by sex (males: β = -0.0126, 95% CI -0.0892, 0.0640; females: β = 0.0322, 95% CI -0.0367, 0.1011). By performing age-stratified subgroup analysis, the association also varied by age and sex. Males aged 20–30 years presented a negative trend (β = -0.0570, 95% CI -0.1656, 0.0517), and males with the age of 31–40 years showed a positive trend (β = 0.0810, 95% CI -0.0312, 0.1931). Women showed a positive trend by age (females of 20–30 years: β = 0.0051, 95% CI -0.0935, 0.1036; females of 31–40 years: β = 0.0265, 95% CI -0.0767, 0.1296). In race-stratified subgroup analysis, the relations varied by sex and race. To be specific, non-Hispanic black males showed a negative trend (β = -0.0754, 95% CI -0.2695, 0.1188), and males of other races exhibited a positive trend. The trend was positive for women of all races.

**Conclusion:**

Differences were detected in the association between serum LDL-C/Apo B ratio and lumbar BMD among cases aged 20–40 years across sex, age, and race/ethnicity. In addition, the inflection points in U-shaped relationships were also calculated.

**Supplementary Information:**

The online version contains supplementary material available at 10.1186/s12891-023-06325-w.

## Introduction

Osteoporosis (OP) displays the features of bone mass loss, bone quality impairment, and a higher risk of fracture among males and females. It shows association with high risk of complications and mortality [1, 2], and exerts a serious impact on its socioeconomic burden. Therefore, special attention should be paid to modifiable risk factors such as smoking, alcohol consumption, decreased body mass index, physical inactivity and poor nutrition [3]. In the past, the management for cases showing the above characteristics has emphasized the identification of secondary factors that lead to decreased bone mass and the treatment of fractures by pain control and orthopedic procedures [4]. Due to the measurements of bone mineral density (BMD), improved treatment skills and increased awareness among the population, OP has emerged as the primary disorder with several manifestations, which can be managed through aggressive prevention and treatment. Great progress has been made in understanding the complicated pathogenic mechanisms of the disease [5, 6]. Consensus has been reached on the relationship strength of reduced BMD with the risk of fracture and on the significance of bone quality as another risk factor for fracture [7].

Recently, some scholars have proposed that bone is an endocrine organ that regulates metabolic homeostasis by releasing bone-specific peptides [8]. A number of bone diseases, including OP, are associated with metabolic changes, and increasing attention has been focused on the close relationship between lipid metabolism and bone metabolism. It is known that cholesterol is related to bone metabolism, and vitamin D, a critical metabolite, exerts an important effect on maintaining bone calcification. Hypercholesterolemia has also been shown to induce bone loss [9]. Cholesterol delivery induced by low-density lipoprotein cholesterol (LDL-C) obviously enhances osteoclast viability, while LDL-C depletion inhibits osteoclast formation [10]. Apolipoprotein B (Apo B) represents a vital part of lipid metabolism. A high plasma Apo B level is an unfavorable factor of atherosclerosis [11], it can be used as a routine lipid test to assess atherosclerosis risk [12]. In addition, cases of cardiovascular disease have also been shown to be associated with an increased risk of OP [13]. Atherosclerotic lesions affect not only peripheral blood vessels but also intraosseous arterioles [14]. Besides, patients with atherosclerosis are also more vulnerable to OP [15]. This may be ascribed to the decreased bone turnover rate caused by bone microvascular disease [16]. All of the aforementioned studies suggest a correlation between cholesterol metabolism, atherosclerosis and OP. Based on the ability of Apo B to assess atherosclerosis risk, Apo B may be an indicator to assess OP. The LDL-C/Apo B ratio has been identified to be the valid proxy of LDL particle size [17], which has been contained as an influencing factor in several studies, including the Framingham Heart Disease Study [17–19]. Currently, conclusions regarding relation between LDL-C and BMD are inconsistent, and studies on relation between LDL-C/Apo B ratio and BMD are also lacking. Therefore, this study focused on investigating relation between LDL-C/Apo B ratio and lumbar BMD among young adults through National Health and Nutrition Examination Survey (NHANES).

## Methods

### Study population

The NHANES refers to a research program for assessing adult and child health and nutrient statuses in the USA beginning in the early 1960s. It is a survey of different populations or health topics. The survey is conducted annually in a representative random sample of approximately 5,000 people in the United States. These people are located in counties throughout the United States, and 15 counties are included each year. The database includes dietary data, demographic data, laboratory data (the data was obtained through laboratory testing and included complete blood count with a 5-part differential haematology instrument, cholesterol, triglycerides, oral glucose tolerance test, alkaline phosphatase levels, blood urea nitrogen levels, total calcium levels, etc.), examination data (​the data was obtained by instrumental measurements and included blood pressure, overall bone density, lung capacity, muscle strength and grip strength tests, etc.), limited access data and questionnaire data. The results can be applied to determine the incidence of major disease and the associated risk factors. Our study was a cross-sectional study and the inclusion and exclusion criteria were as follows: **Inclusion criteria**: 1. Subjects in three NHANES cycles from 2011 to 2016 (because both Apo B and lumbar BMD data were collected during the above cycle) and 2. Aged 20–40 years. **Exclusion criteria**: 1. Patients with lumbar BMD score deficiency; 2. Patients with LDL-C deficiency; 3. Patients with APO B deficiency; 4. Patients with cancer; and 5. Patients taking lipid-lowering drugs. We finally obtained data on 2,027 young adults between the ages of 20 and 40. Our study used open data from NHANES database and included human participants. In compliance with the Declaration of Helsinki, the approval of all NHANES protocols was obtained from the Ethics Review Board of the National Center for Health Statistics. Moreover, all the subjects provided informed consent (NCHS IRB/ERB Protocol Number: 2011–2012: Protocol #2011–17; 2013–2014: Continuation of Protocol #2011–17; 2015–2016: Continuation of Protocol #2011–17). Detailed information is available at https://www.cdc.gov/nchs/nhanes/irba98.htm.

### Variables

In the current work, LDL-C/Apo B ratio was selected to be the exposure variable. Lumbar BMD was selected to be the outcome variable. The rest are covariates.

#### LDL-C/Apo B ratio measure

Serum Apo B levels were detected by a Seimens ProSpec Analyzer. Meanwhile, serum LDL-C levels were determined based on high-density lipoprotein cholesterol (HDL-C), triglyceride (TG) and total cholesterol (TC) levels measured by Friedewald calculation [20]. Both Serum Apo B and LDL-C levels were expressed in mg/dL. Serum TC, TG and HDL-C levels were detected by the Roche/Hitachi Modular P Chemistry Analyzer.

#### Lumbar BMD measure

Lumbar BMD was evaluated by a qualified radiologist. The Hologic Discovery Model A densitometer (Hologic, Inc., Bedford, Massachusetts) was applied in scanning the outcome variable lumbar BMD, whereas the Apex 3.2 software was adopted for analysis.

#### Covariates and confounders

Covariates and confounders included age, sex, education, race/ethnicity, waist circumference (WC), income-to-poverty ratio, drinking, smoking, calcium supplementation, physical activity, serum uric acid, serum alkaline phosphatase (ALP), total protein, TC, TG, HDL-C, serum calcium, serum phosphorus, fasting blood glucose (FBG), diastolic blood pressure (DBP) as well as systolic blood pressure (SBP). Detailed data on variables can be found in the NHANES guidelines and manual at http://www.cdc.gov/nchs/nhanes/.

Measurements and classifications for all variables are shown in Table S1.

### Statistical analysis

The weights of all estimates were determined in the NHANES samples to further reflect sample population features [21, 22]. Weighted chi-square test (χ2) was employed to investigate categorical data, whereas continuous variables were investigated with the use of a weighted linear regression model. Relations of serum LDL-C/Apo B ratio with lumbar BMD in different sex groups were explored using weighted multiple stratified linear regression, while the smooth curve fitting model was utilized for analyzing the nonlinear relation between serum LDL-C/Apo B ratio and lumbar bone mineral density in different sex groups with different ages and races/ethnicities. Concerning the nonlinear relationship, the inflection point was calculated by saturation threshold analysis. Later, the weighted two-piecewise linear regression model was constructed.

All data were analyzed using EpowerStats (http://www.empowerstats.com) and R software package (http://www.R-project.org). In addition, the significance level was determined at *P* < 0.05.

## Results

Table 1 presents the baseline features of 2027 patients. In relative to serum LDL-C/Apo B ratio quintile 1 group, TG levels gradually decreased, while TC and serum calcium levels gradually elevated with the increase of LDL-C/Apo B ratio.

**Table 1:** Weighted characteristics of the participants based on serum LDL-C/Apo B ratio quintiles

**serum LDL-C/Apo B ratio**	**Total**	**Q1**	**Q2**	**Q3**	**Q4**	**Q5**	***P* value**
Age (years)	29.82 ± 6.13	29.50 ± 6.13	29.25 ± 6.16	29.48 ± 6.20	29.31 ± 6.08	30.16 ± 6.10	0.2048
Sex (%)							0.8217
Male	53.43	51.56	55.48	54.06	53.54	55.21	
Female	46.57	48.44	44.52	45.94	46.46	44.79	
Race/ethnicity (%)							0.0145
Non-Hispanic white	35.96	51.62	58.30	53.72	60.24	59.07	
Non-Hispanic black	18.75	11.86	11.84	13.53	12.32	15.40	
Mexican American	15.19	16.13	13.33	14.41	10.18	7.37	
Other race/ethnicity	30.09	20.38	16.53	18.34	17.27	18.16	
Level of education (%)							0.0016
Less than high school	17.37	21.39	15.49	15.15	12.50	10.96	
High school	21.16	22.89	20.12	20.36	20.23	19.46	
More than high school	61.47	55.72	64.39	64.50	67.27	69.58	
Income to poverty ratio	2.24 ± 1.54	2.35 ± 1.50	2.46 ± 1.58	2.50 ± 1.58	2.42 ± 1.58	2.78 ± 1.62	0.0010
Smoking behavior (%)							0.0039
Every day	16.77	18.06	18.54	19.77	14.39	12.60	
Some days	5.62	5.93	6.53	6.65	4.05	4.54	
Not at all	14.01	16.49	19.53	15.23	16.69	12.57	
Not recorded	63.59	59.52	55.41	58.35	64.87	70.29	
Alcohol consumption (%)							0.3376
High alcohol use	11.69	13.39	11.25	11.18	10.69	12.55	
None/moderate alcohol use	70.35	73.37	75.38	71.53	74.17	68.81	
Not recorded	17.96	13.24	13.37	17.29	15.14	18.64	
Physical activity (%)							0.0216
Sedentary	5.77	4.42	6.67	5.60	6.04	4.88	
Low activity	14.85	14.78	15.78	18.18	14.13	14.62	
Moderate activity	22.10	22.52	21.47	25.32	20.97	27.02	
High activity	3.95	2.72	6.43	5.15	2.05	4.30	
Not recorded	53.33	55.57	49.65	45.75	56.81	49.18	
Waist circumference (cm)	94.15 ± 16.72	98.65 ± 18.60	94.65 ± 16.65	95.73 ± 17.12	93.46 ± 16.32	92.47 ± 13.98	< 0.0001
Total protein (g/dL)	7.22 ± 0.45	7.14 ± 0.44	7.14 ± 0.44	7.25 ± 0.44	7.19 ± 0.41	7.17 ± 0.43	0.0009
Total cholesterol (mg/dL)	182.47 ± 36.42	166.73 ± 32.99	176.26 ± 35.27	180.58 ± 34.18	186.30 ± 33.97	195.86 ± 36.99	< 0.0001
Triglycerides (mg/dL)	107.96 ± 67.89	159.98 ± 89.14	118.14 ± 63.77	98.03 ± 50.72	89.39 ± 43.79	76.61 ± 35.04	< 0.0001
HDL-C (mg/dL)	52.47 ± 14.23	47.71 ± 15.69	50.29 ± 14.06	53.04 ± 14.81	53.51 ± 11.97	58.01 ± 12.80	< 0.0001
Fasting blood glucose (mg/dL)	93.07 ± 24.37	97.20 ± 31.39	92.63 ± 23.15	92.44 ± 21.52	89.79 ± 9.13	89.61 ± 14.46	< 0.0001
systolic blood pressure	115.25 ± 12.72	118.77 ± 14.07	115.00 ± 12.09	115.05 ± 11.52	114.30 ± 12.04	114.05 ± 10.69	< 0.0001
diastolic blood pressure	68.31.25 ± 11.39	69.75 ± 11.80	67.54 ± 11.54	68.44 ± 9.91	67.55 ± 10.64	67.97 ± 10.64	0.0282
Serum uric acid (mg/dL)	5.37 ± 1.35	5.53 ± 1.46	5.32 ± 1.26	5.44 ± 1.35	5.33 ± 1.27	5.37 ± 1.33	0.1566
Serum total alkaline phosphatase (IU/L)	62.55 ± 19.45	66.65 ± 31.55	62.24 ± 18.53	62.14 ± 18.70	61.16 ± 17.21	60.32 ± 17.60	0.0004
Serum phosphorus (mg/dL)	3.74 ± 0.56	3.73 ± 0.58	3.76 ± 0.59	3.74 ± 0.53	3.72 ± 0.54	3.78 ± 0.59	0.5329
Serum calcium (mg/dL)	9.36 ± 0.31	9.31 ± 0.31	9.33 ± 0.28	9.37 ± 0.32	9.39 ± 0.31	9.41 ± 0.31	< 0.0001
Calcium supplementation (%)							0.0090
Not use	86.33	89.64	83.01	84.91	85.52	81.18	
< 0.4 g/d	7.94	6.59	8.80	8.51	10.36	9.63	
≥ 0.4 g/d	5.72	3.77	8.19	6.58	4.12	9.19	
Lumbar BMD (g/cm2)	1.04 ± 0.14	1.05 ± 0.16	1.02 ± 0.14	1.05 ± 0.14	1.04 ± 0.13	1.05 ± 0.13	0.0128

Mean ± SD for continuous variables: the* P* value was calculated by the weighted linear regression model

Percent (%) for categorical variables: the *P* value was calculated by the weighted chi-square test

*Abbreviations*: *Apo B* Apolipoprotein B, *BMD* Bone mineral density, *HDL-C* High density lipoprotein cholesterol, *LDL-C* Low density lipoprotein cholesterol

Tables 2 and 3 display diverse multiple linear regression model analyses of male and female young adults, respectively. Covariates from Table 1 were adjusted, and the relation between serum LDL-C/Apo B ratio and lumbar BMD varied by sex (males: β = -0.0126, 95% CI -0.0892, 0.0640; females: β = 0.0322, 95% CI -0.0367, 0.1011). Based on age-stratified subgroup analysis, the association also varied by age and sex. Males aged 20–30 years presented a negative trend (β = -0.0570, 95% CI -0.1656, 0.0517), while males with the age of 31–40 years showed a positive trend (β = 0.0810, 95% CI -0.0312, 0.1931). Women showed a positive trend by age (females aged 20–30 years: β = 0.0051, 95% CI -0.0935, 0.1036; females aged 31–40 years: β = 0.0265, 95% CI -0.0767, 0.1296). As shown in race-stratified subgroup analysis, the relations varied by sex and race. Specifically, non-Hispanic black males showed a negative trend (β = -0.0754, 95% CI -0.2695, 0.1188), and males of other ethnicities presented a positive trend. The trend was positive for women of all races (Tables 2 and 3).

**Table 2:** The correlation between serum LDL-C/Apo B ratio and lumbar BMD (g/cm2) in males (*n* = 1083)

	Model 1 β (95% CI)	Model 2 β (95% CI)	Model 3 β (95% CI)
LDL-C/Apo B ratio	0.0363 (-0.0203, 0.0930)	0.0130 (-0.0421, 0.0681)	-0.0126 (-0.0892, 0.0640)
Stratified by year			
20–30 years	-0.0208 (-0.1004, 0.0588)	-0.0371 (-0.1139, 0.0397)	-0.0570 (-0.1656, 0.0517)
31–40 years	0.1040 (0.0233, 0.1846)	0.0739 (-0.0059, 0.1536)	0.0810 (-0.0312, 0.1931)
Stratified by race			
Non-Hispanic White	0.0159 (-0.0772, 0.1090)	0.0153 (-0.0778, 0.1085)	0.0240 (-0.1137, 0.1616)
Non-Hispanic Black	-0.1232 (-0.2724, 0.0260)	-0.1138 (-0.2629, 0.0353)	-0.0754 (-0.2695, 0.1188)
Mexican American	0.0693 (-0.0413, 0.1798)	0.0678 (-0.0442, 0.1798)	0.0462 (-0.1350, 0.2274)
Other race/ethnicity	0.0666 (-0.0278, 0.1610)	0.0665 (-0.0281, 0.1611)	0.0051 (-0.1205, 0.1306)

Model 1, no covariates were adjusted

Model 2, age, race/ethnicity were adjusted

Model 3, age, race/ethnicity, education, income to poverty ratio, waist circumference, smoking behavior, alcohol consumption, physical activity, calcium supplementation, serum total alkaline phosphatase, serum uric acid, total protein, total cholesterol, triglycerides, HDL cholesterol, serum phosphorus, serum calcium, fasting blood glucose, systolic blood pressure and diastolic blood pressure were adjusted. In the subgroup analysis stratified by age and race/ethnicity, the model was not adjusted for age or race/ethnicity

**Table 3:** The correlation between serum LDL-C/Apo B ratio and lumbar BMD (g/cm2) in females (*n* = 944)

	Model 1 β (95% CI)	Model 2 β (95% CI)	Model 3 β (95% CI)
LDL-C/Apo B ratio	0.0202 (-0.0319, 0.0723)	0.0095 (-0.0417, 0.0608)	0.0322 (-0.0367, 0.1011)
Stratified by year			
20–30 years	0.0144 (-0.0589, 0.0878)	0.0055 (-0.0670, 0.0780)	0.0051 (-0.0935, 0.1036)
31–40 years	0.0240 (-0.0505, 0.0985)	0.0089 (-0.0643, 0.0821)	0.0265 (-0.0767, 0.1296)
Stratified by race			
Non-Hispanic White	0.0144 (-0.0712, 0.1000)	0.0067 (-0.0795, 0.0929)	0.0300 (-0.0919, 0.1518)
Non-Hispanic Black	-0.0775 (-0.2062, 0.0511)	-0.0759 (-0.2046, 0.0528)	0.0346 (-0.1323, 0.2015)
Mexican American	-0.0524 (-0.1844, 0.0796)	-0.0478 (-0.1782, 0.0826)	0.0122 (-0.1933, 0.2177)
Other race/ethnicity	0.0945 (0.0075, 0.1814)	0.0967 (0.0093, 0.1842)	0.1065 (-0.0078, 0.2208)

Model 1, no covariates were adjusted

Model 2, age, race/ethnicity were adjusted

Model 3, age, race/ethnicity, education, income to poverty ratio, waist circumference, smoking behavior, alcohol consumption, physical activity, calcium supplementation, serum total alkaline phosphatase, serum uric acid, total protein, total cholesterol, triglycerides, HDL cholesterol, serum phosphorus, serum calcium, fasting blood glucose, systolic blood pressure and diastolic blood pressure were adjusted. In the subgroup analysis stratified by age and race/ethnicity, the model was not adjusted for age or race/ethnicity

**Fig. 1:**
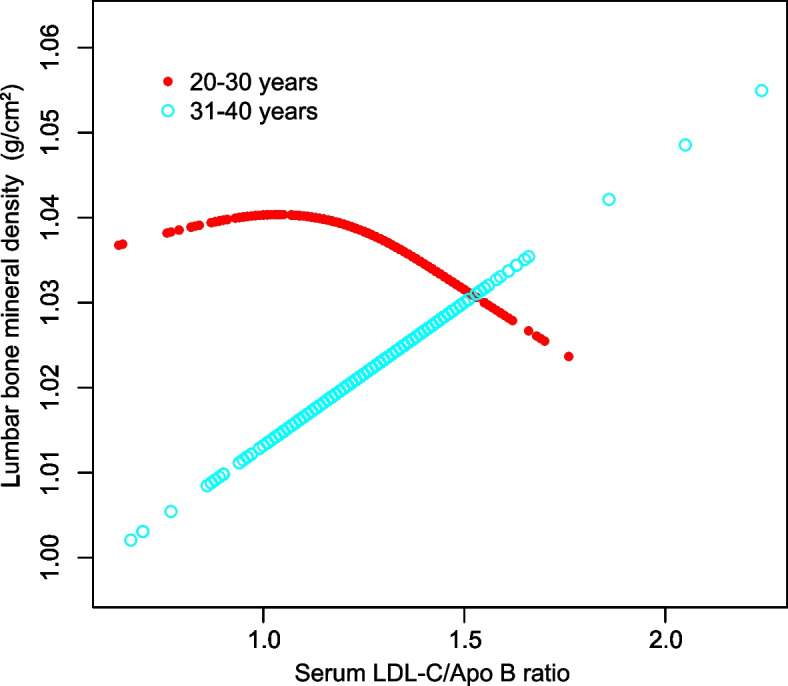
The correlation between serum LDL-C/Apo B ratio and lumbar BMD in males stratified by age. (Race/ethnicity, education, income to poverty ratio, waist circumference, smoking behavior, alcohol consumption, physical activity, calcium supplementation, serum total alkaline phosphatase, serum uric acid, total protein, total cholesterol, triglycerides, HDL cholesterol, serum phosphorus, serum calcium, fasting blood glucose, systolic blood pressure and diastolic blood pressure were adjusted.)

**Fig. 2:**
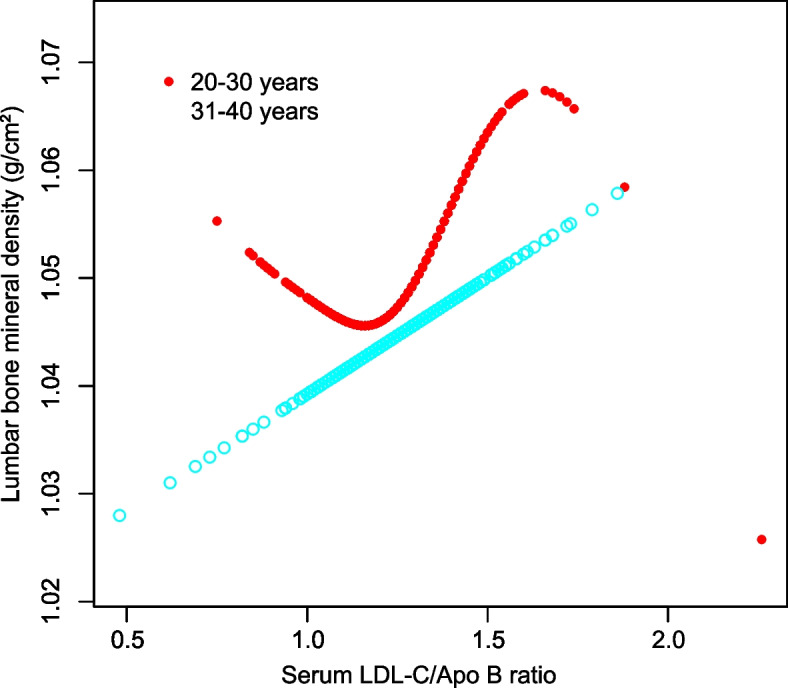
The correlation between serum LDL-C/Apo B ratio and lumbar BMD in females stratified by age. (Race/ethnicity, education, income to poverty ratio, waist circumference, smoking behavior, alcohol consumption, physical activity, calcium supplementation, serum total alkaline phosphatase, serum uric acid, total protein, total cholesterol, triglycerides, HDL cholesterol, serum phosphorus, serum calcium, fasting blood glucose, systolic blood pressure and diastolic blood pressure were adjusted.)

**Fig. 3:**
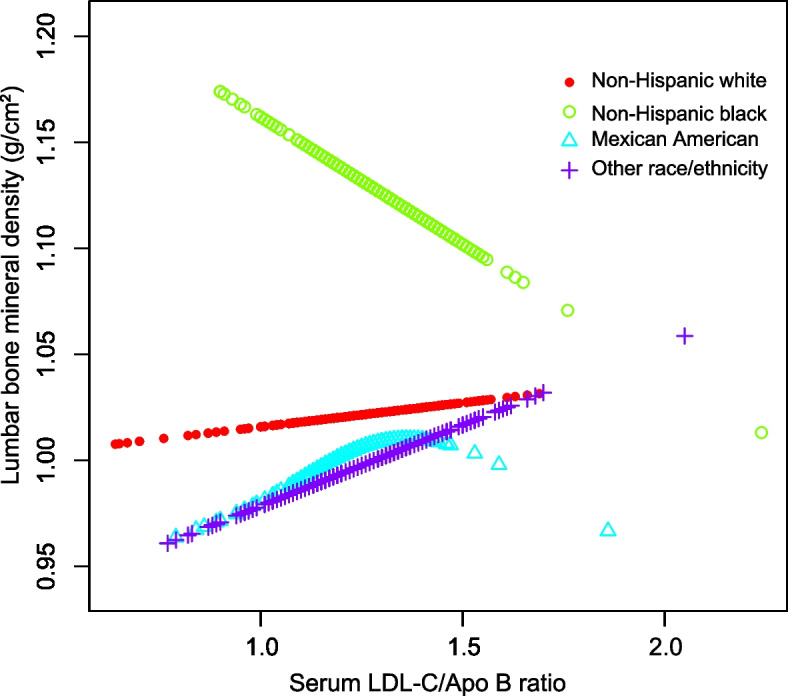
The correlation between serum LDL-C/Apo B ratio and lumbar BMD in males stratified by race/ethnicity. (Age, education, income to poverty ratio, waist circumference, smoking behavior, alcohol consumption, physical activity, calcium supplementation, serum total alkaline phosphatase, serum uric acid, total protein, total cholesterol, triglycerides, HDL cholesterol, serum phosphorus, serum calcium, fasting blood glucose, systolic blood pressure and diastolic blood pressure were adjusted.)

**Fig. 4:**
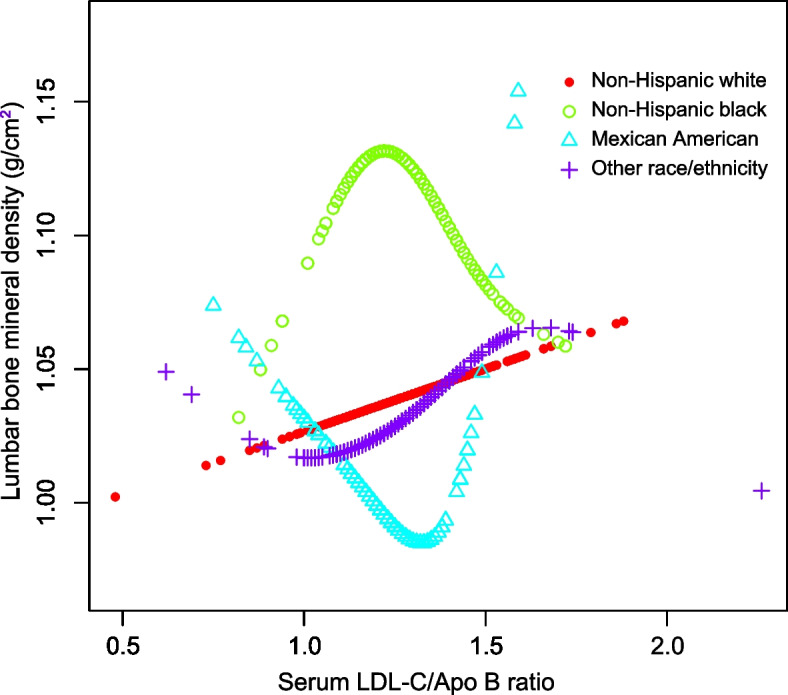
The correlation between serum LDL-C/Apo B ratio and lumbar BMD in females stratified by race/ethnicity. (Age, education, income to poverty ratio, waist circumference, smoking behavior, alcohol consumption, physical activity, calcium supplementation, serum total alkaline phosphatase, serum uric acid, total protein, total cholesterol, triglycerides, HDL cholesterol, serum phosphorus, serum calcium, fasting blood glucose, systolic blood pressure and diastolic blood pressure were adjusted.)

In addition, smooth curve fitting was conducted with the aim of investigating the potential nonlinear correlation between serum LDL-C/Apo B ratio and lumbar BMD (Figs. 1, 2, 3 and 4). Therefore, there existed a nonlinear connection between serum LDL-C/Apo B ratio and lumbar BMD among males and females aged 20–30 years, non-Hispanic black women, Mexican Americans (regardless of the sex), and women of other races/ethnicities. Among them, men aged 20–30 years, Mexican American men, and non-Hispanic black women showed an inverted U-shaped relationship. Mexican American women displayed a U-shaped relationship. The inflection point was further calculated to be 1.08 males aged 20–30 years, 1.39 for Mexican American males, 1.44 for Mexican American women, and 1.20 for non-Hispanic black women (Table 4).

**Table 4:** Threshold effect analysis of serum LDL-C/Apo B ratio on lumbar BMD

Lumbar bone mineral density	Adjusted β (95% CI)
**Male**	
20-30 years	
Fitting by standard linear model	-0.0570 (-0.1656, 0.0517)
Fitting by two-piecewise linear model	
Inflection point	1.08
serum LDL-C/Apo B ratio < 1.08	0.0096 (-0.2967, 0.3159)
serum LDL-C/Apo B ratio > 1.08	-0.0697 (-0.1915, 0.0520)
Log-likelihood ratio	0.638
Mexican American	
Fitting by standard linear model	0.0462 (-0.1350, 0.2274)
Fitting by two-piecewise linear model	
Inflection point	1.39
serum LDL-C/Apo B ratio < 1.39	0.2113 (-0.0101, 0.4327)
serum LDL-C/Apo B ratio > 1.39	-0.4082 (-0.8121, -0.0044)
Log-likelihood ratio	0.007
**Female**	
Non-Hispanic black	
Fitting by standard linear model	0.0346 (-0.1323, 0.2015)
Fitting by two-piecewise linear model	
Inflection point	1.20
serum LDL-C/Apo B ratio < 1.20	0.4646 (0.0779, 0.8514)
serum LDL-C/Apo B ratio > 1.20	-0.1111 (-0.3140, 0.0917)
Log-likelihood ratio	0.009
Mexican American	
Fitting by standard linear model	0.0122 (-0.1933, 0.2177)
Fitting by two-piecewise linear model	
Inflection point	1.44
serum LDL-C/Apo B ratio < 1.44	-0.1290 (-0.3472, 0.0892)
serum LDL-C/Apo B ratio > 1.44	1.7282 (0.6076, 2.8488)
Log-likelihood ratio	< 0.001

Age, race/ethnicity, education, income to poverty ratio, waist circumference, smoking behavior, alcohol consumption, physical activity, calcium supplementation, serum total alkaline phosphatase, serum uric acid, total protein, total cholesterol, triglycerides, HDL cholesterol, serum phosphorus, serum calcium, fasting blood glucose, systolic blood pressure and diastolic blood pressure were adjusted. In the subgroup analysis stratified by age and race/ethnicity, the model was not adjusted for age or race/ethnicity

## Discussion

In this study on young adults aged 20–40 years in the United States, it was found that (1) there was a sex difference in the correlation between serum LDL-C/Apo B ratio and lumbar BMD, and (2) the association also varied with age and race/ethnicity.

Approximately 80% of cholesterol synthesis occurs in the liver and intestines, with the remaining 20% occurring in bone cells [23]. Lipid rafts play a crucial role in signal transduction during osteoclastogenesis, and cholesterol is an important component of lipid rafts [10]. On the other hand, studies have also confirmed that excessive cholesterol accumulation may increase bone renewal. The outcome can be the promotion of osteoclast formation and the subsequent loss of bone mass [24]. Lipoproteins may also affect osteoclast activity by regulating cholesterol levels. LDL-C has been reported to substantially increase osteoclast activity by inducing cholesterol delivery, whereas LDL-C consumption inhibits osteoclast formation [25]. All the above studies indicate that cholesterol is closely related to bone metabolism, but the underlying mechanism is not completely clear.

Hyperlipidemia is usually involved in the occurrence and progression of diseases (or chronic complications) [16]. The LDL-C/Apo B ratio represents the proxy of LDL particle size, with a decreased LDL-C/Apo B ratio suggesting a low LDL-C density within LDL particles and higher risks of infiltration into the artery wall and atherosclerosis [26]. According to numerous studies, LDL-C/Apo B ratio is superior to Apo B alone in the predication of atherosclerosis. A previous study shows that bone microvascular disease may represent an important pathogenic mechanism underlying the declined bone turnover [27]. A histological study reports that in addition to the peripheral vessels, atherosclerotic lesions also develop in the intraosseous arterioles during atherosclerosis [14]. Another study indicates that the bone mineral density of the crus without atherosclerotic plaque is higher than that of the crus with atherosclerotic plaque [15]. All of these studies demonstrate that the LDL-C/Apo B ratio reflects atherosclerosis severity and OP degree.

However, conclusions on the connection of serum LDL-C and APO B with BMD are inconsistent, and research regarding the association of serum LDL-C/APO B ratio with BMD is also lacking. According to a cross-sectional study including 481 participants, serum LDL-C may not show association with BMD [28]. In a study of the Old Order Amish population, LDL-C is inversely related to BMD, and it is also observed that the Apo B R3500Q variant predicts lower BMD levels in the whole body, lumbar spine and femoral neck [29]. According to a study conducted more than a decade ago, serum LDL-C shows a positive relation with BMD [30]. Another study indicates a nonlinear relation of serum LDL-C with lumbar BMD among postmenopausal women [31]. Apart from sex, age, and race/ethnicity, differences in genetic risk factors, obesity status, metabolic status, and lifestyle habits (e.g., smoking, alcohol consumption, exercise) may also be a possible explanation for the variability. A study evaluating LDL-C/Apo B ratio within arteriosclerosis demonstrates that cases showing a low LDL-C/Apo B ratio had a higher number of low-density LDL particles, which exhibits an increased TG level [32]. Consistently, our research found that TG content increased within the lower quintile of serum LDL-C/Apo B ratio in comparison with the higher quintiles. A recent study suggests that the reduced LDL-C/Apo B ratio can be related to lower bone turnover among type 2 diabetes mellitus cases independently. However, no relation of LDL-C/Apo B ratio with BMD was observed in this study [33]. Subgroup analyses were conducted based on STROBE guidelines [34] in this study. Therefore, there were differences in relation between serum LDL-C/Apo B ratio and lumbar BMD by age, sex and race/ethnicity. In addition, we found that this relationship in males aged 20–30 years, Mexican American men and non-Hispanic black women showed an inverted U-shaped relationship, and that Mexican American women had a U-shaped relationship. The inflection point was also calculated to be 1.08 for males aged 20–30 years, 1.39 for Mexican American men, 1.44 for Mexican American women, and 1.20 for non-Hispanic black women. These differences might be attributed to age, gender, and racial/ethnic heterogeneity.

## Strengths and limitations

First, we perform a subgroup analysis based on the STROBE statement [34]. Furthermore, a weighted, multiracial, typical sample was applied in this study to ensure the high representativeness of our results. A nonlinear relationship was observed by using smooth curve fitting. Nevertheless, certain limitations should be noted. At first, the cross-sectional design was used. Therefore, causality between serum LDL-C/Apo B ratio and lumbar BMD in the young adult population could not be explored. Second, tumor patients and people taking lipid-lowering drugs were excluded from the current work, aiming to eliminate the potentially remarkable impact on serum LDL-C/Apo B ratio and lumbar BMD. Therefore, our study was not representative of these populations. Third, to generalize the findings, our study did not rule out other diseases that might affect bone health. Fourth, some of the covariate information collected through the questionnaire may have a recall bias. Finally, due to some limitations of the NHANES database, we did not include hip/neck femur BMD, T scores or Z scores in our study, so this paper has some limitations.

## Conclusions

In summary, there were differences in the connection between serum LDL-C/Apo B ratio and lumbar BMD among cases aged 20–40 years across sex, age, and race/ethnicity. The inflection points in U-shaped relationships were calculated. Moreover, these studies may provide some reference for further revealing the underlying mechanism between serum LDL-C/Apo B ratio and OP.

## Supplementary Information

**Additional file 1: Supplementary Table S1.** Detailed information on variables.

## Data Availability

The data used in our study is publicly available. All data utilized and/or analyzed in the present study can be obtained from 2011–2016 NHANES at http://www.cdc.gov/nchs/nhanes/.

## Abbreviations

OP: Osteoporosis
Apo B: Apolipoprotein B
BMD: Bone mineral density
LDL-C: Low-density lipoprotein cholesterol
ESC/EAS: European Society of Cardiology/European Atherosclerosis Association
NHANES: National Health and Nutrition Examination Survey
HDL-C: High-density lipoprotein cholesterol
TG: Triglycerides
TC: Total cholesterol
WC: Waist circumference
ALP: Alkaline phosphatase
FBG: Fasting blood glucose
SBP: Systolic blood pressure
DBP: Diastolic blood pressure
