# Supplementary material for: The correlation between the serum LDL-C/Apo B ratio and lumbar bone mineral density in young adults
Source: BMC Musculoskelet Disord. 2023 Mar 22;24:213. doi: 10.1186/s12891-023-06325-w (PMC10031959; doi:10.1186/s12891-023-06325-w)
Supplement: Supplementary file 1 — Additional file 1: Supplementary Table S1. Detailed information on variables. [file 12891_2023_6325_MOESM1_ESM.docx]

**Supplementary Table S1** Detailed information on variables

| **Variables** | **Measures** | **Grouping for adjustment** |
| --- | --- | --- |
| Age (years) | Information on gender, age, race/Hispanic origin, education, and income was obtained from NHANES participants through interviews. | ​​The subgroup analysis was divided into two categories: 20-30 years and 31-40 years. |
| Sex | Same as above | Male and Female |
| Race/ethnicity | Same as above | Non-Hispanic white, Non-Hispanic black, Mexican American and Other race/ethnicity |
| Level of education | Same as above | Less than high school, High school and More than high school |
| Income to poverty ratio | Same as above | / |
| Smoking behavior | Information on smoking, alcohol consumption and exercise was obtained through questionnaire interviews and quality control was conducted accordingly. | Every day, Some days, Not at all and Not recorded |
| Alcohol consumption | Same as above | High alcohol use, None/moderate alcohol use and Not recorded |
| Physical activity | The NHANES database collects physical activity through a global Physical activity questionnaire. Participants reported their frequency of and time spent in vigorous and moderate work activity, walking or bicycling and vigorous or moderate recreational activities. Metabolic equivalent of task (MET) scores were assigned for each activity based on NHANES guidelines. | Sedentary(≤1.5 METs), Low activity(1.6-2.9 METs), Moderate activity(3-5.9 METs), High activity（≥6 METs）and Not recorded |
| Waist circumference | Waist circumference data were obtained by anthropometry | / |
| Total protein | Venous blood samples require fasting of at least nine hours, but no more than 24. Blood samples are processed, stored and shipped to the Collaborative Laboratory Service for analysis. All operations follow the Laboratory Procedures Manual. | / |
| Total cholesterol | Same as above | / |
| Triglycerides | Same as above | / |
| HDL-C | Same as above | / |
| Fasting blood glucose | Same as above | / |
| systolic blood pressure | Blood pressure data is obtained by measuring with a sphygmomanometer | / |
| diastolic blood pressure | Blood pressure data is obtained by measuring with a sphygmomanometer | / |
| Serum uric acid | Venous blood samples require fasting of at least nine hours, but no more than 24. Blood samples are processed, stored and shipped to the Collaborative Laboratory Service for analysis. All operations follow the Laboratory Procedures Manual. | / |
| Serum total alkaline phosphatase | Same as above | / |
| Serum phosphorus | Same as above | / |
| Serum calcium | Same as above | / |
| Calcium supplementation | Measurements of supplementation are included in the Nutrition Assessment section of the NHANES database. The nutritional assessment component of the current NHANES includes a 24-hour dietary recall interview for participants of all ages. | Not use, <0.4g/d and ≥0.4g/d |
| Lumbar BMD (g/cm2) | The Hologic Discovery Model A densitometer (Hologic, Inc., Bedford, Massachusetts) was applied in scanning the outcome variable lumbar BMD, whereas the Apex 3.2 software was adopted for analysis. | / |
| serum LDL-C/Apo B ratio | ​Serum Apo B levels were detected with a Seimens ProSpec Analyzer. Serum TC, TG and HDL-C levels were detected using a Roche/Hitachi Modular P Chemistry Analyzer. Serum LDL-C levels were determined based on high-density lipoprotein cholesterol (HDL-C), triglyceride (TG) and total cholesterol (TC) levels measured by Friedewald calculation. | ​The subgroup analysis is divided into five categories based on numerical values: Q1, Q2, Q3, Q4, Q5. |

Abbreviation: Apo B, apolipoprotein B; BMD, bone mineral density; HDL-C, high density lipoprotein cholesterol; LDL-C, low density lipoprotein cholesterol.

Detailed data on variables can be found in the NHANES guidelines and manual at http://www.cdc.gov/nchs/nhanes/.​
